# The Effect of a Theory of Planned Behavior-based Educational Intervention on Sexual and Reproductive Health in Iranian Adolescent Girls: A Randomized Controlled Trial

**Published:** 2017-11-28

**Authors:** Fatemeh Darabi, Mohammad Hossein Kaveh, Farideh Khalajabadi Farahani, Mehdi Yaseri, Fereshteh Majlessi, Davoud Shojaeizadeh

**Affiliations:** ^1^ Department of Public Health, Asadabad School of Medical Sciences, Asadabad, Iran; ^2^ Department of Health Education and Health Promotion, School of Public Health, Kermanshah University of Medical Sciences, Kermanshah, Iran; ^3^ Department of Health Education and Health Promotion, School of Health, Shiraz University of Medical Sciences, Shiraz, Iran; ^4^ Department of Population, Health and Family Planning, National Institute for Population Research, Tehran, Iran; ^5^ Department of Epidemiology and Biostatistics, Tehran University of Medical Sciences, Tehran, Iran; ^6^ Department of Health Education and Health Promotion, School of Public Health, Tehran University of Medical Sciences, Tehran, Iran

**Keywords:** Adolescent, Reproductive health, Education, Theory of planned behavior

## Abstract

**Background:** We aimed to assess the effect of a theory of planned behavior (TPB)-based educational intervention on attitude, norms, parental control, behavioral control, and intention in high school girls in Tehran, Iran.

**Study design:** Randomized controlled trial.

**Methods:** This study was conducted among 578 high school girls, 12 to 16 yr, in Tehran, Iran in 2016. The subjects were randomly assigned to the experimental (n=289) and control (n=289) groups using multistage random cluster sampling. TPB is the basis for both education and evaluation; therefore, the TPB-based questionnaire was used before and after the intervention. The intervention included three months education and six months follow up. The obtained data were analyzed using SPSS version 16 through statistical tests and analysis of covariance.

**Results:** Significant improvement in attitude (difference=16.8; 95% CI: 15.3, 18.3), subjective norms (16.4; 95% CI=14.83 to 18.11), perceived behavioral control (18.0; 95% CI: 16.6, 19.4), perceived parental control (17%; 95% CI: 15.1, 18.9), behavioral intention (18.4%; 95 CI: 14.8, 18.3), and behavior (18.5; 95% CI:16.8, 20.2) was observed in experimental group compared to control group (P<0.001).

**Conclusions:** Theory-based educational intervention in sexual and reproductive health can effectively reduce the high-risk behaviors related to sexual and reproductive health in adolescent girls. Health and education policy-makers are advised to review the current education programs and replace them with new influential education programs related to sexual and reproductive health in the school system.

## Introduction

Sexual and reproductive health (SARH) is an essential basis for the economic development of communities^1^. The consequences of early pregnancy and sexually transmitted infections (STIs), including HIV/AIDS, threaten the health of people in the second decade of life more than any other age group^2^. Notwithstanding, adolescents are the greatest hope to alter the situation against STIs, AIDS, and early pregnancy^3^.

Iran is a young country so that around‏ half of the population is under the age of 27yr^4^. Young people aged 10-29 yr constitute approximately 39% of the population in Iran^5^. Hence, more attention is needed to enhance their SARH from young ages in school^3^. The HIV/AIDS epidemic is spreading in Iran^4^ and HIV-infected people were estimated to be 106,000 in 2014^6^. Adolescents aged 20-29 yr and 25-34 yr respectively account for 20% and 38% of all Iranian HIV-infected people^4^.

The mode of transmission in Iran is shifting from needle sharing to sexual routes, nevertheless, the sexually active unmarried adolescents normally do not receive the necessary information and services to prevent from STIs, HIV/AIDS infection, unintended pregnancy, and unsafe abortion, so they become vulnerable to these problems‏. In Iran, there is no comprehensive information regarding such high-risk behaviors and no study so far has‏ particularly looked at the ways of protection and predictors of that high-risk behaviors^6^.

The transmission of HIV/STIs through sexual contact has increased in Iran, highlighting the importance of sexual and reproductive health education^5^. However, few studies provide sexual and reproductive health education to adolescents. Indeed, adolescents are uninformed about sexual and reproductive health^7^ which is probably due to the lack of education and information provided by families and schools. Poor knowledge, fear, and anxiety foster the negative attitudes toward sexual and reproductive health^8^, indicating the need to design the education programs which address those problems^9^.

The effectiveness of a school-based HIV/STIs prevention interventions is reported ^10, 11^. Due to the increases in high-risk behaviors like sexual relationships among Iranian adolescents, there is a necessary need to hold HIV/AIDS, and sexual and reproductive health education programs for high-risk groups. Meanwhile, preventive education programs should be formally incorporated into the school curriculum for high school students^12^. Young girls as future mothers ought to acquire knowledge concerning SARH as well as useful and requisite skills leading to a safe and healthy sexual and reproductive life^13^.

The effectiveness of an educational intervention depends on an appropriate application of behavioral science theories^14^. TPB is one of the principal theories used to design the evidence-based interventions. TPB assumes that attitude, subjective norms, and perceived behavioral control lead to the development of a behavioral intention and so the behavioral intention is the immediate antecedent of behavior^15^.

**Figure 1:**
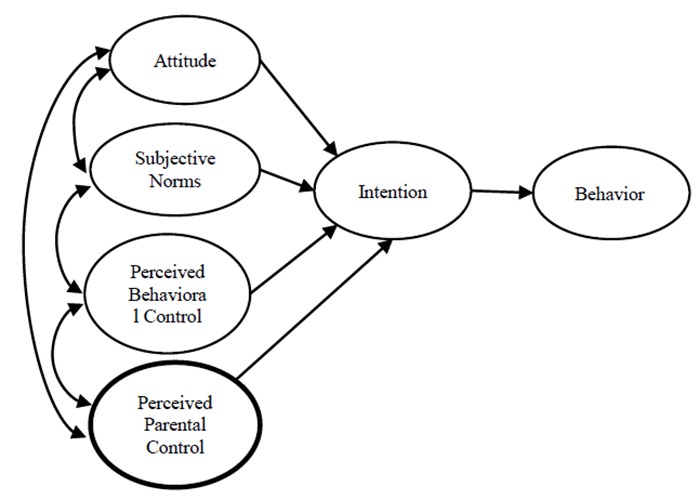
The modified theory of planned behavior

We investigated the role of parental control on students’ perception by TPB^14^. In some developing countries, parents prefer to use resources providing information about SARH ^15, 16^. In the current study, we added perceived parental control to TPB as an additional construct (Figure 1). In Iran, there is a huge gap in assessing the effectiveness of TPB-based education programs on attitude, norms, parental control, and behavioral control and intention among high school girls.

## Methods

### Study setting & populations

This randomized controlled trial (RCT) study was conducted among first-year high school girls, 12-16 yr, residing in Tehran, Iran, who voluntarily participated in the study.

The informed written consent was taken before the study from both students and their parents or guardians. The study was approved by Ethics Committee of Tehran University of Medical Sciences (ethics code No. 651, dated Monday, Apr 25, 2016) and registered in Iran Clinical Trials Registry (IRCT2015070623089N2 code).

The sample size calculation was based on knowledge as it needs more samples compared to behavior score. Thereby, to detect 10 score difference in the improvement of the scores between two groups with a power of 90%, considering the standard deviation of knowledge scores of 22.9 and 25.2 ,^17^, and assumed design effect of 2.1 and 10% loss to follow up, at least 285 samples needed for each group. Ultimately, 289 students in each group entered into the study.

### Sampling & Randomization

**Figure 2:**
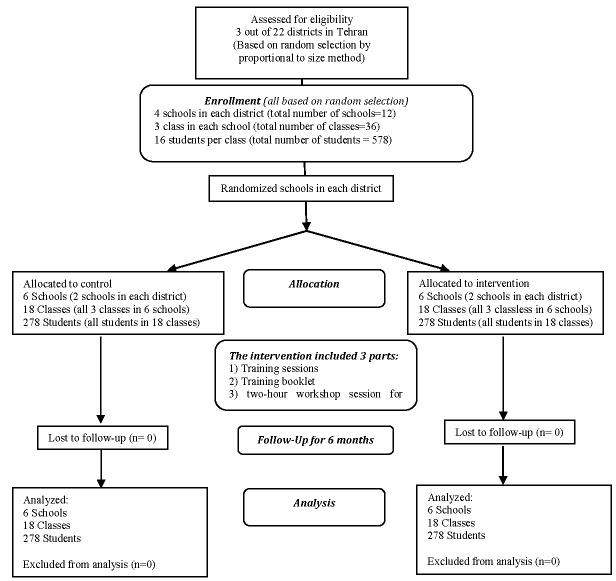
Flowchart of sampling procedure for the Interventional study

Three out of 22 districts in Tehran were initially selected using proportional stratified random sampling method. In every district, four schools were randomly selected using probability-proportional-to-size sampling method. In each school, three classes and in each class, 16 students were randomly recruited. In every district, two schools were randomly assigned to the control group and two schools for the experimental group. We used the same approach in the permuted block randomization with the block length of 2 and 4. Thus, we could be certain of having an equal number of schools (two schools) in each district. Figure 2 demonstrates the process of participants enrollment in the groups. The inclusion criteria were being the first-year high school girl, living in Tehran, volunteer to participate, and having parent's written consent. The exclusion criteria were lack of participation in the intervention program, migration, and incomplete questionnaire (Figure 2).

### Intervention program

The training program was a two-part school-based approach, training sessions with a booklet for adolescents, a two-hour workshop about high-risk behaviors, and sexual as well as reproductive health-related issues for parents. The approach was based on a premise that to change the behaviors putting adolescents at risk, they need to acquire precise information regarding puberty health, adolescent health, STD diseases, HIV transmission routes, HIV prevention, and an understanding of how high-risk behaviors make them vulnerable and the necessary skills to make healthy choices.

The experimental approach with a variety of communication techniques was used to encourage adolescents to participate in the intervention. The results of the pilot study (i.e., lack of knowledge about sexual and reproductive health) were the only helpful information obtained to design the current study. We used educational strategies to determine the behavioral goals in each of the learning areas including knowledge section (presentation classes (40-45 min), lectures, and slideshow), attitude section (role-playing, training animation, story-writing, and teasers brainstorming), subjective norms section (training sessions for parents, and booklet), perceived behavioral control section (story-writing, small group discussion, and booklet), and perceived parental control section (training sessions for parents).

A booklet was developed on a middle-school reading level, adapted for adolescents aged 12-16 yr. In addition, the final draft of the booklet was evaluated by adolescents outside the schools before applying to the current study. The full-color booklet with 17 standard-size pictorial pages entitled “Training 12-16-yr-old Adolescents at a Turning Point in Life.” addressing adolescents and shedding light on their importance (puberty, menstrual health, and HIV/STD). The introductory section outlined the content of the booklet. The other sections were based on the TPB model constructs using simple language. During the sessions, topics in the booklet were discussed, and feedback was solicited from the students. Six months after the intervention, students were followed up. Overall, the training program was held in four sessions (in two 45-min parts with a 15-min break). After the study, training materials such as booklet were given to the control group. The whole training program lasted for three months.

### Measures

A self-administered questionnaire with various dimensions including nutrition, physical activity, adolescent health, menstrual health, and HIV was developed using the direct measures of TPB. A structured anonymous questionnaire was used as the main data collection instrument. Some parts of this instrument were founded on WHO questionnaire^18^. The WHO questionnaire involves questions about sexual and reproductive health knowledge and its sources, sexual attitudes, sexual behavior, reproductive health services, and SARH outcomes^19^. Another part of the instrument was developed by literature review and a qualitative study together with eight focus group discussions (FGDs) with 40 participants. Each FGD lasted one hour.

The questionnaire was structured in three parts as follows:

**Part A:** The demographic information‏ including age, father's level of education, mother’s level of education, mother's job, father’s job, and economic status.
**Part B:** General knowledge about sexual and reproductive health: for example, “HIV is transmitted through unprotected vaginal and anal sex” (28 items rated as ‘‘true,’’ ‘‘false,’’ ‘‘don’t know’’).
**Part C:** Modified TPB section

The participants completed a 132-item questionnaire measuring knowledge by six constructs of the TPB model (attitude, perceived behavioral control, perceived parental control, behavioral intention, behavior, and subjective norms.

Twenty-one‏ items assessed respondents’ attitudes towards people with AIDS, for example, “In my opinion, sexual and reproductive health is a serious problem for the health of all people.”

Nineteen‏ items were phrased to reflect social norms, for example, “People around me think that I should avoid risky behaviors (like unprotected sex, direct injection of contaminated blood, and tattoos) which result in sexual and reproductive health.”

Twenty-five‏ items were phrased to reflect perceived behavioral control, for example, “I'm sure that I can‏ avoid risky behaviors such as unprotected sex and tattoos, which transmit infections to one another.”

Behavioral intention toward sexual and reproductive health was addressed by eighteen items, for example, “I'm going to talk about sexual and reproductive health problems with my parents and family members in the future.”

Eight‏ item addressed participants’ perceived parental control (in conceptual framework section) about sexual and reproductive health, for example, “my parents give me enough training and guidance on the subject of sexual and reproductive health.”

This study added an additional construct namely perceived parental control to the original theory which might influence sexual and reproductive health behavior and has been taken into consideration before. Parents play a critical role in the creation of knowledge and positive attitude of their children’s regarding sexual and reproductive health behaviors^15^.

Finally, eight items were used to evaluate sexual and reproductive health skills. All items were scored on a five-point Likert-type scale (ranging from “strongly disagree” to “strongly agree”). To make the results comparable with other scales, we transformed scores from 0 to 100 with the following formulation: New score = 100 * (score – 1) / 4.

In our study, the minimum possible score for each question was one and range of scores was four (=5-1). The total scores for each subscale were calculated by averaging the scores of all questions on that scale, ranging from 0 to 100. To obtain the score of each dimension, the mean scores of all items in that dimension was obtained. Of course, this procedure is exchangeable (one could first get the mean scores, and then using the mentioned transformation).

Negative items were reversely coded before computing the mean scores of scale. It took 40 min to complete the questionnaires in pre-post intervention. The questionnaires were completed after explanation of the purpose of the study and voluntary consent.

To assess the validity of the scale, we assessed face validity, content validity, and construct validity. To assess the reliability of the scale, the internal consistency and stability of the scale were measured. In the qualitative face validity, participants expressed no problems with reading and understanding the items. The numeric value of content validity ratio (CVR) was 0.65, determined by the Lawshe table. We used an ordinal scale with four possible responses to obtain content validity index (CVI) for relevance, simplicity, and clarity of each item. The mean CVI was 0.78. The responses ranged from 1= not relevant to 4= very relevant. The number of those judging the items as relevant or clear was determined by the content experts.

The constructed questionnaire was evaluated for validity by exploratory and confirmatory factor analysis (EFA & CFA), and reliability was assessed by Cronbach’s alpha coefficient. The confirmatory factor analysis (CFA) indicated a good fit to the data. Internal consistency was evaluated by Cronbach’s α coefficient. Cronbach’s α coefficient of 0.7 or above was considered satisfactory. Moreover, a sub-sample of students (n = 45) completed the questionnaire twice with a 2-week interval to examine the stability of the scale by calculating Intra-class Correlation Coefficient (ICC) where the ICC of 0.4 or above was considered acceptable (Table 1).

**Table 1:** Number of items, and α of Iranian students' sexual and reproductive health questionnaire constructs (n=578)

**Subscales**	**No. of items**	**Mean**	**SD**	**Alpha Cronbach**
Attitudes towards sexual & reproductive health	21	24.88	9.91	0.96
Subjective Norm	19	23.80	11.26	0.97
Behavioral Intention	18	25.66	9.40	0.96
Perceived parental control	8	25.45	13.02	0.96
Perceived behavioral control	25	24.57	9.98	0.96
Behavior	13	26.96	11.34	0.95
Total	104	25.04	4.40	0.86

### Data analysis

Data were analyzed using SPSS, version 23 (Chicago, IL, USA). To make the results comparable, the scores of the TPB constructs (1 to 5) were converted to scores from 0 to 100. To consider the correlation of measurements in the clusters, multilevel analysis was used. The Likelihood Ratio (LR) test was used to acquire the proper number of levels in multilevel analysis. The comparison of the baseline values between the two groups was performed by a two-level multilevel analysis. The three-level multilevel analysis was used to evaluate the changes in each group. To compare the changes in the scores of the dimensions between two groups, the interaction between the groups and time in a multilevel analysis was examined.‏ To assess the deterministic role of the educational intervention in behavior improvement, while adjusting, the role of other significant factors like knowledge was controlled and some other multilevel analyses using the different set of explanatory variables were used. At long last, we simultaneously used multivariate analysis of variance (MANOVA) to evaluate the effect of education on improving all dimensions. All statistical tests were two-sided and *P*-values less than 0.05 were considered statistically significant.

## Results

Demographic characteristics of participants are presented in Table 2. Preliminary analysis revealed no significant difference in the baseline characteristics, such as demographic variables, knowledge, and the TPB model constructs, between the participants in the control and experimental groups.

**Table 2:** Baseline Demographic and socioeconomic characteristics of experimental students (289 experimental and 289 control groups)

**Variables**	**Control**		**Experimental**		***P*** **value**
	**n**	**%**	**n**	**%**	
**Father’s level of education (yr)**					0.291
<6	23	8.1	34	11.8	
6-12	175	61.4	191	66.1	
>12	87	30.5	64	22.1	
**Mother’s level of education (yr)**					0.427
<6	37	12.9	35	12.1	
6-12	175	61.0	204	70.6	
>12	75	26.1	50	17.3	
**Father’s employment status**					0.297
Employed	269	93.1	275	95.2	
Unemployed	20	6.9	14	4.8	
**Mother’s employment status**				0.637
Employed	79	27.3	74	25.6	
Housewife	210	72.7	215	74.4	
**Economic status (self-reported)**				0.442
Very good	23	8.0	12	4.2	
Good	128	44.3	114	39.4	
Average	123	42.6	131	45.3	
Weak	15	5.2	32	11.1	

A total of 578 students participated in the study. The mean age of participants was 14.1 (±1). 366 (63.8%) of fathers and 397 (65.8%) of mothers had below diploma education. Other demographic characteristics are summarized in Table 2.

Table 3 depicts the distribution of the baseline, follow-up, and change in the knowledge, TPB constructs, and comparison of the groups. The model analysis indicated that the improvement in attitude, perceived behavioral control, perceived parental control, behavioral intention, behavior, and subjective norms was significantly higher in the experimental group compared to control group six months after the training intervention (all *P*<0.001, based on the interaction between the groups and time in a multilevel analysis).

**Table 3:** Group differences in knowledge and the TPB constructs at baseline and 6-month follow-up

**Variables**	**Intervention**		**Control**		**Diff. (95% CI)**	***P*** **value**
	**Mean**	**SD**	**Mean**	**SD**		
Knowledge						
Baseline	49.0	19.9	49.0	19.9	-0.3 (-5.0, 4.4)	0.901
Follow-up	85.3	11.0	55.3	20.8	30.1 (27.3, 32.8)	0.001
*P*-value	0.001		0.001			
Attitude						
Baseline	55.8	6.8	56.4	7.0	-0.6 (-2.2, 1.0)	0.472
Follow-up	93.3	6.3	76.6	11.2	16.8 (15.3, 18.3)	0.001
*P*-value	0.001		0.001			
Subjective norms					
Baseline	58.7	8.7	58.7	8.6	0.1 (-2.0, 2.1)	0.939
Follow-up	93.9	7.3	77.5	12.2	16.4 (14.8, 18.1)	0.001
*P*-value	0.001		0.001			
Perceived Behavioral control				
Baseline	62.2	9.1	61.9	9.2	0.3 (-1.9, 2.5)	0.786
Follow-up	94.9	5.0	77.0	10.9	18.0 (16.6, 19.4)	0.001
*P*-value	0.001		0.001			
Perceived parental control				
Baseline	56.5	11.0	53.7	11.1	2.8 (-0.01, 5.6)	0.053
Follow-up	93.1	8.6	76.1	14.0	17.0 (15.1, 18.9)	0.001
*P*-value	0.001		0.001			
Behavioral Intention				
Baseline	63.6	10.2	63.8	9.9	-0.2 (-2.6, 2.2)	0.869
Follow-up	94.2	6.0	75.7	10.9	16.4 (14.8, 18.3)	0.001
*P*-value	0.001		0.001			
Behavioral						
Baseline	64.1	9.9	63.1	10.4	1.0 (-1.4, 3.4)	0.416
Follow-up	93.1	7.2	74.6	12.7	18.5 (16.8, 20.2)	0.001
*P*-value	0.001		0.001			

The analysis on structure of the model indicated significantly higher improvement in attitude (Difference: 16.8; 95% CI: 15.3, 18.3), subjective norms (16.4; 95% CI: 14.8, 18.0), perceived behavioral control (18.0; 95% CI: 16.6, 19.4), perceived parental control (17.0, 95% CI: 15.1, 18.9), behavioral intention (16.4; 95% CI: 14.8, 18.0), and behavior (18.5, 95% CI: 16.8, 20.2) in the experimental group compared to the control group (based on the interaction between the groups and time in a multilevel analysis). According to the MANOVA, the constructs of TPB model for reproductive health exhibited significant improvement in the experimental group compared to the control group (*P*<0.001 for all dimensions).

**Figure 3:**
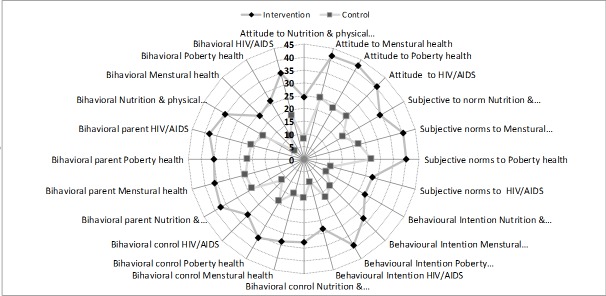
changes each of structure based on the dimensions theory of planned behavior

All dimensions dramatically improved in the experimental group compared to the control group (Figure 3).

### Effects of other constructs and variables on the behavior score change

After adjusting the effect of baseline behavior on knowledge, attitude, subjective norms, behavioral control, behavioral parent, and behavioral intention in a multilevel regression analysis, the experimental group got statistically significant higher score than the control group (3.79; 95% CI: 1.34, 6.24, *P*=0.003). Moreover, knowledge (*P*<0.001), subjective norms (*P*=0.029), behavioral parent (*P*<0.001), and behavioral intention (*P*<0.001) had statistically significant effect on the sexual and reproductive health behavior score in this model (Table 4).

**Table 4:** Results of linear multilevel regression analysis on the effect of TPB construct to predict of sexual and reproductive health behavior based on multilevel analysis

**Group**	**B (95% CI)**	***P*** **value**
Ref.	3.79 (1.36, 6.22)	0.003
Behavioral (Pretest)	0.25 (-0.92, 0.51)	0.491
Knowledge	1.96 (1.42, 2.53)	0.001
Attitude	0.47 (-0.41, 1.43)	0.313
Subjective Norms	0.95 (0.11, 1.83)	0.029
Behavioral control	0.56 (-0.43, 1.52)	0.257
Behavioral parent	1.39 (0.74, 2.11)	0.001
Behavioral Intention	1.81 (0.92, 2.74)	0.001

B: Regression coefficient

## Discussion

Our results indicated that this three-month intervention was influential and significantly increased the sexual and reproductive health knowledge and behaviors. The pre-intervention baseline survey showed that students’ knowledge, attitude, subjective norms, perceived behavioral control, behavioral intention, and behavior about sexual and reproductive health was inadequate. Significant changes in total variables conclude that school-based education is useful and practical on changing sexual and reproductive health behaviors in adolescents after 6-month follow-up. The TPB-based educational intervention improved all dimensions including nutrition and physical activity, menstrual health, puberty health, and HIV/AIDS, however not to the same extent. The scores of behavioral intention and behavior were significantly more than the scores of attitude and subjective norms after the intervention, possibly due to the time required for the intervention to be more effective. The continuity of such an educational intervention can help to increase attitude and subjective norms^14^.

In this study, sexual and reproductive health behavior rates increased from 64.1 to 93.1 in the experimental group, after the educational intervention. The effect of educational intervention on improvement of sexual and reproductive health performance showed regular SARH might reduce frailty, especially in individuals at higher risk of disability. A school environment is a suitable place for the education of sexual and high-risk behaviors prevention. Therefore, it is necessary to design and implement programs in schools for students for SARH improvement.

The results of our study indicated a statistically significant difference in the scores of attitude in experimental group before and after the educational intervention, in consistent with another study^2^ on predictors of adoption and maintenance of sexual health and prevention of high-risk behaviors in a community sample. Attitude towards an important issue such as sexual and reproductive health can be‏ imagined regarding perceived sensitivity.‏ This attitude is a critical factor which encourages and motivate people to‏ adopt preventive behaviors, hence, the education programs should be allocated as a part of adolescents' activities to raise perceived vulnerability. Therefore, it is necessary for intervention planners to rely on the fundamental change in people’s attitude by effective methods that motivate healthy behaviors^20^.

In line with some studies^14, 21^, subjective norms significantly increased in our study. However, another study did not confirm a significant increase in subjective norms due to the intervention^22^. In the context of future interventions, in addition to the usual methods, the peer group approach can be used to facilitate the learning process. Peer group education approach is a practical strategy for behavior change. It is undeniable that the greatest impact, positive or negative, is on the performance of young people in all aspects. Besides, there is an opportunity for adolescents that can provide and convey the necessary knowledge about any subject using interactive and participatory values such as group discussions, workshops, role-playing, and etc. Talking about sexual and reproductive health should be taken by parents. In fact, both parents and children are reluctant to talk about the sexual and reproductive health-related issues.‏ In 2003, a qualitative study was conducted in the south of Iran, using Focus Group Discussions (FGDs), and demonstrated that the most preferred individuals to educate family planning issues to adolescents are the health staff, teachers and parents, respectively^2^. Therefore, the social norms as a necessary factor in the health behavior changes and improvement should be considered while designing a school-based intervention.

The mean score of perceived behavioral control was also examined in our study and indicated a significant increase in the experimental group. Perceived behavioral control, self-efficacy included, for experimental group significantly increased after the educational intervention, which is consistent with the results of different studies ^14,23^. Self-efficacy is the most robust structural in the prediction of behavior. So, the self-efficacy change occurs after the active participation of individuals to maintain healthy behavior, and usually, people who show the remarkable change in behavior have higher self-efficacy levels for the particular behavior. Therefore, it is necessary to consider environmental factors outside the control, for example, lack of enough time, being embarrassed in front of parents while talking about sexual issues, or the attitude that "I think so rudely and disrespect." To overcome these obstacles, the person-centered interventions seems sufficient but there are environmental barriers such as lack of cost required for a combination of interventions^24^ (individual-environmental).

In addition, the significant increase in the mean score of behavioral intention was observed in the experimental group, emphasizing the effectiveness of the intervention implementation, in line with other studies^25, 26^. In TPB, the behavioral intention is regarded as the paramount behavior predictor. Intentions are as motivational factors affecting behavior and indicating the intensity of individuals' motivation and attempts to perform a task^27^. The more strong behavioral intention, the more probable is to perform a behavior. In fact, the intention is required for behavior but is not sufficient. Educational intervention could positively affect the students' behavioral intention for health promotion.

The findings of this study suggest that parents can play a critical role in sexual and reproductive health behaviors. Parents usually influence their children’s reproductive health behavior through care and control. The parents should try to create and maintain an intimate and good relationship with adolescents and to discuss values and morals in society with adolescents. Families accorded the higher value to the education of their children‏ the lower level of risky behaviors (including; sexual activities, alcohol using, and delinquency) among children ^17,29^. Accordingly, the existence of closer relationships between parents and children by creating a positive family environment may influence high-risk behaviors as well as sexual and reproductive health behaviors^34^; confirming the results of other studies,,^14, 19, 29^. Moreover, consistent with other studies ^28, 30^, the present study indicated the significant mean score of knowledge in the experimental group.

In this study, the mean scores of the control group slightly increased in all studied constructs. Since there are some kinds of reproductive health-related training in high schools in Iran, it was not unexpected to see such an improvement in the control group. Furthermore, the questions in the pre-test phase aroused curiosity about these items among students in the long term. In various studies^5, 31 ,32^, significant changes have reported for the control group. In the current study, one cannot rule out the possible role of the large sample size in determining the statistical significance of changes in the control group.

The effect of education on the TPB model on reproductive health behavior and behavioral intention scores was effective (Tables 3 and 4), concordant with the findings of the studies^25, 33^. The present study demonstrated that the school-based sexual and reproductive health education could be a promising program for enhancing adolescent SARH. This model could be scaled up to the national level for improving adolescent sexual and reproductive health. Besides, this study has the potentials to contribute to the development and implementation of programs at national levels and in countries with similar situations.

Although this study has some limitations, the study was implemented in urban areas and among adolescent girls. Thus, we can only generalize the results to adolescent girls inhabiting in urban areas of Tehran. They could not be generalized to other geographical areas due to cultural, social, and economic differences. Moreover, these findings are based on the students’ self-reporting. Furthermore, the study was conducted among high-school girls with the mean age of 14; therefore, the results do not suit the younger and older people. Moreover, this study was carried out only among female students in state schools and could not be generalized to adolescent boys. Future intervention studies should evaluate the role of different factors like psychological factors (including peers, self-esteem, and self-control) conducting on other competing academic requirements and must work more closely with parents, teachers, advisors and other community-based organization in an attempt to address the problems in school.

Despite the limitations, this study benefited from some strengths including face-to-face education, having a control group, and use of booklet to reduce attrition. It is suggested to conduct studies on both genders. Studies should also be carried out based on other health training models in order to find the most practical and valid model for conducting studies in this field. The results of this study could be used by the department of education, adolescent health units, adolescent counseling centers, and schools.

## Conclusions

The theory-based educational intervention has an effective role to improve the first-year high school girls’ positive thinking. These programs have to be carried out on the basis of factors contributing to sexual and reproductive health behaviors among girls, and efficient solutions have to be adapted to control them, leading to lower levels of high-risk behaviors. Additionally, all of the TPB constructs played a pivotal role in improving the students’ sexual and reproductive health behavior.

## Acknowledgements

The authors would like to thank all of the participants and schools’ personnel.

## Conflict of interest statement

No conflict of interest is declared.

## Funding

No funding was received.

## Highlights

- Sexual and reproductive health behaviors are the key factor in female adolescent health.
- Sexual and reproductive health behaviors in adolescence can be improved by appropriate intervention.
- Theory of planned behavior (TPB) can provide suitable intervention framework to improve sexual and reproductive health behaviors in female students.

## References

[1] Lupton D (2015). Quantified sex: a critical analysis of sexual and reproductive self-tracking using apps. ‎Cult Health Sex.

[2] Chandra-Mouli V, Svanemyr J, Amin A, Fogstad H, Say L, Girard Fo (2015). Twenty years after International Conference on Population and Development: where are we with adolescent sexual and reproductive health and rights?. J Adolesc Health.

[3] Vakilian K, Mousavi SA, Keramat A (2014). Estimation of sexual behavior in the 18-to-24-years-old Iranian youth based on a crosswise model study. BMC Research Notes.

[4] Tavoosi A, Zaferani A, Enzevaei A, Tajik P, Ahmadinezhad Z (2004). Knowledge and attitude towards HIV/AIDS among Iranian students. BMC Public Health.

[5] Farahani FK, Akhondi MM, Shirzad M, Azin A (2017). Hiv/sti risk-taking sexual behaviours and risk perception among male university students in tehran: Implications for hiv prevention among youth. J Biosoc Sci.

[6] Shamsipour M, Khajehkazemi R, Haghdoost AA, Setayesh H, KarimanMajd S, Mostafavi E (2012). Knowledge, Attitude, and Practice of Clerical Students with Respect to HIV/AIDS in Iran, 2011. J Religion Health.

[7] Maki PM, Rubin LH, Valcour V, Martin E, Crystal H, Young M (2015). Cognitive function in women with HIV Findings from the Women's Interagency HIV Study. Neurology.

[8] Montaner JSG, Lima VD, Harrigan PR, Louren‏أ§o L, Yip B, Nosyk B (2014). Expansion of HAART coverage is associated with sustained decreases in HIV/AIDS morbidity, mortality and HIV transmission: the HIV Treatment as Prevention a experience in a Canadian setting. PloS One.

[9] Van Lunsen R, van Dalen L (2004). Determinants of sexual and reproductive behavior European J Contracept. Reprod.

[10] Durojaye E (2009). Realizing Access to Sexual Health Information and Services for Adolescents Through the Protocol to the African Charter on the Rights of Women. Wash & Lee J Civil Rts & Soc Just.

[11] Wong LP, Chin CK, Low WY, Jaafar N (2008). HIV/AIDS-related knowledge among Malaysian young adults: Findings from a nationwide survey. J Int AIDS Soc.

[12] Bashirian S, Barati M, Mohammadi Y, Mostafaei H (2016). Factors associated with hookah use among male high school students: the role of demographic characteristics and hookah user and non-user prototypes. J Res Health Sci.

[13] Sawyer SM, Afifi RA, Bearinger LH, Blakemore S-J, Dick B, Ezeh AC (2012). Adolescence: a foundation for future health. Lancet.

[14] Taghdisi MH, Babazadeh T, Moradi F, Shariat F (2016). Effect of educational intervention on the fruit and vegetables consumption among the students: applying theory of planned behavior. J Res Health Sci.

[15] Mbonile L, Kayombo EJ (2008). Assessing acceptability of parents/guardians of adolescents towards introduction of sex and reproductive health education in schools at Kinondoni Municipal in Dar es Salaam city. East Afr J Public Health.

[16] Sayles JN, Macphail CL, Newman PA, Cunningham WE (2010). Future HIV vaccine acceptability among young adults in South Africa. Health Educ Behav.

[17] Bashirian S, Hidarnia A, Allahverdipour H, Hajizadeh E (2012). Application of the theory of planned behavior to predict drug abuse related behaviors among adolescents. J Res Health Sci.

[18] Cleland J, Verrall J, Vaessen M (2006). Preferences for the sex of children and their influence on reproductive behavior. Demography.

[19] HBearinger L, ESieving R, Ferguson J, Sharma V (2007). Global perspectives on the sexual and reproductive health of adolescents: patterns, prevention, and potential. Lancet.

[20] Sharma M (2007). International school‐based interventions for preventing obesity in children. Obes Rev.

[21] Webb TL, Sheeran P (2006). Does changing behavioral intentions engender behavior change? A meta-analysis of the experimental evidence. Psychol.

[22] Rhodes RE, Courneya KS (2003). Investigating multiple components of attitude, subjective norm, and perceived control: An examination of the theory of planned behaviour in the exercise domain. Br J Soc.

[23] Wang S, Fan J, Zhao D, Yang S, Fu Y (2016). Predicting consumers’ intention to adopt hybrid electric vehicles: using an extended version of the theory of planned behavior model. Transportation.

[24] Johnson DB, Beaudoin S, Smith LT, Beresford S, LoGerfo JP (2004). Increasing fruit and vegetable intake in homebound elders: the Seattle Senior Farmers’ Market Nutrition Pilot Program. Prev Chronic Dis.

[25] Barati M, Allahverdipour H, Moinei B, Farhadinasab A, Mahjub H (2011). Evaluation of theory of planned behavior-based education in prevention of MDMA (ecstasy) use among university students. Medical Journal of Tabriz University of Medical Sciences.

[26] Jin NP, Lee S, Lee H (2015). The effect of experience quality on perceived value, satisfaction, image and behavioral intention of water park patrons: new versus repeat visitors. Int J Tourism Res.

[27] Kothe EJ, Mullan B, Butow P (2012). Promoting fruit and vegetable consumption Testing an intervention based on the theory of planned behavior. Appetite.

[28] Donnelly JE, Blair SN, Jakicic JM, Manore MM, Rankin JW, Smith BK (2009). American College of Sports Medicine Position Stand Appropriate physical activity intervention strategies for weight loss and prevention of weight regain for adults. Med Sci Sports Exerc.

[29] Bilal SM, Spigt M, Dinant GJ, Blanco R (2015). Utilization of Sexual and Reproductive Health Services in Ethiopia–Does it affect sexual activity among high school students?. Sex Reprod Healthc.

[30] Farahani FKA, Alikhani S, Mohammadi MR, Bahonar A (2007). Parents’ attitudes towards adolescent boy's reproductive health needs and practice in Tehran. Iran J Psychiatr.

[31] Jahangiry L, Shojaeizadeh D, Farhangi MA, Yaseri M, Mohammad K, Najafi M (2015). Interactive web-based lifestyle intervention and metabolic syndrome: findings from the Red Ruby (a randomized controlled trial). Trials.

[32] Sanavi FS, Navidian A, Rakhshani F, Ansari-Moghaddam A (2014). The effect of education on base the Theory of Planned Behavior toward normal delivery in pregnant women with intention elective cesarean. Bimonthly Journal of Hormozgan University of Medical Sciences.

[33] Pooreh S, Nodeh ZH (2015). Impact of education based on theory of planned behavior: an investigation into hypertension-preventive self-care behaviors in Iranian girl adolescent. Iran J Public Health.

[34] Farahani FKA, Cleland J, Mehryar AH (2011). Associations between family factors and premarital heterosexual relationships among female college students in Tehran. Int Perspect Sex Reprod Health.

